# Variations in Pulmonary Fissure: A Source of Collateral Ventilation and Its Clinical Significance

**DOI:** 10.7759/cureus.23121

**Published:** 2022-03-13

**Authors:** Asha Joshi, Pragatisheel Mittal, Arpita M Rai, Ranjana Verma, Bharti Bhandari, Shyama Razdan

**Affiliations:** 1 Anatomy, Government Institute of Medical Sciences, Greater Noida, IND; 2 Anatomical Sciences, Government Institute of Medical Sciences, Greater Noida, IND; 3 Anatomy, Hamdard Institute of Medical Sciences & Research, New Delhi, IND; 4 Physiology, Government Institute of Medical Sciences, Greater Noida, IND

**Keywords:** horizontal lung fissure, oblique lung fissure, cadaver, bronchopulmonary segment, collateral ventilation

## Abstract

Introduction: Oblique and horizontal fissures divide the lungs into lobes. Assessing the incompleteness or absence of fissures is important when planning any surgical procedure in this region. This study aimed to investigate the incidence of variations in the fissures and their implication in clinical practice.

Methods: The sample consists of 70 formalin-fixed lungs (32 right and 38 left lungs). These lungs were assessed for complete, incomplete, and absent fissures and any variations in the fissures on the sternocostal and mediastinal surfaces.

Results: Oblique fissure was incomplete on the sternocostal surface in 18.75% and 21% and on the mediastinal surface in 25% and 21% on the right and left lungs, respectively. Additionally, it was absent in 10.5% of the left lung samples. The horizontal fissure was incomplete in 12.5% on both surfaces and was absent in 25% on the right lung samples, of which 50% had no oblique fissure. No accessory fissure was seen in any of the lungs.

Conclusion: Incomplete and absent fissures can be observed in the right as well as the left lung, suggesting the source of collateral ventilation. This study imparts important information to pulmonologists doing bronchoscopic lung volume reduction therapy or bronchopulmonary segment resection and also to radiologists and anatomists.

## Introduction

The lungs are a pair of vital organs for respiration situated within the thoracic cage on either side of the mediastinum. Each lung is conical in shape and is divided into lobes by double folds of visceral pleura called fissures. Anatomically, the right lung is divided into upper, middle, and lower lobes by oblique and horizontal fissures, and the left lung is divided into upper and lower lobes by an oblique fissure. The right oblique fissure is similar to the left one, although it is less vertical and separates the inferior lobe from the middle and upper lobes. The oblique fissure begins from the upper part of the hilum on the mediastinal surface and cuts the vertebral border at the fourth or fifth level of the thoracic spine. Then, it courses along the costal surface, cuts the inferior border, reappears on the mediastinal surface, and ends at the lower end of the hilum. The horizontal fissure, which is seen only in the right lung, begins at the oblique fissure, courses along the costal surface, cuts the anterior border, appears on the mediastinal surface, and ends at the hilum [1]. Frontal chest radiography can detect horizontal fissures in 60% of cases. The oblique fissure is usually seen via lateral radiography and appears as a curved band from the lateral aspect to the hilum under high-resolution computed tomography (CT) [1-3].

As a result of an inward extension of the visceral pleura, these fissures provide a smooth surface between the lobes. It acts as a plane of cleavage so that during inspiration, the upper part of the lung enlarges forward and laterally, and the lower part of the lung expands downward and backward. Lung fissures may be complete when the lobes remain intact at the hilum by bronchi and pulmonary vessels, but they may also be incomplete when a parenchymal fusion occurs between the lobes [4]. The incomplete fissure may be a source of collateral ventilation, wherein the alveolar structures are ventilated through passages that bypass the normal airways and connect two lung lobes.

In severe emphysema, bronchoscopic lung volume is reduced using one-way valves to attain complete lobar atelectasis, giving an encouraging therapeutic result to improve lung function and quality of life. However, this complete atelectasis cannot be achieved in patients with incomplete fissures because of collateral ventilation [5]. These fissures are also useful in bronchopulmonary segment location, which is significant both anatomically and clinically. Before lobectomy, identifying fissure completeness is important because individuals with incomplete fissures have a higher risk for postoperative air leaks and may require further procedures such as stapling and pericardial sleeves [6]. Furthermore, cardiothoracic surgeons need to fully understand the anatomy of lung fissure variations when planning any surgeries in this region. This knowledge is also extremely important for radiologists to make accurate diagnoses when interpreting radiological images. Most of the studies on variations of the fissures in the cadaveric lung have been done in South India. There is a paucity of similar studies from North India. More so, regional variations in the occurrence of lung fissures have been observed. Hence, considering its anatomical and clinical importance, we aimed to examine the morphology of lung fissures and lobes from cadaveric specimens in North India.

## Materials and methods

The study was a descriptive cross-sectional study done in the Department of Anatomy of Government Institute of Medical Sciences, Greater Noida, Uttar Pradesh, and Hamdard Institute of Medical Sciences in Delhi. Seventy formalin-fixed lungs (32 right lungs and 38 left lungs) stored in the Anatomy museum were used for the study. Ethical clearance was not required as cadavers in the Anatomy department are taken from voluntary body donors for teaching and research purposes under the Anatomy Act.

Inclusion criteria included lungs with intact visceral pleura covering everywhere except at the hilum. Exclusion criteria included the lungs damaged during dissection, lungs that have pathological lesions such as trauma, necrosis, adhesions, etc., and lungs of the cadavers having respiratory or lung pathology as the primary cause of death. Three observers independently observed the presence or absence of major fissures, variations in the major fissures (complete or incomplete), and presence of accessory fissure, if any, and the final conclusion was drawn after thorough discussion.

**Figure 1:**
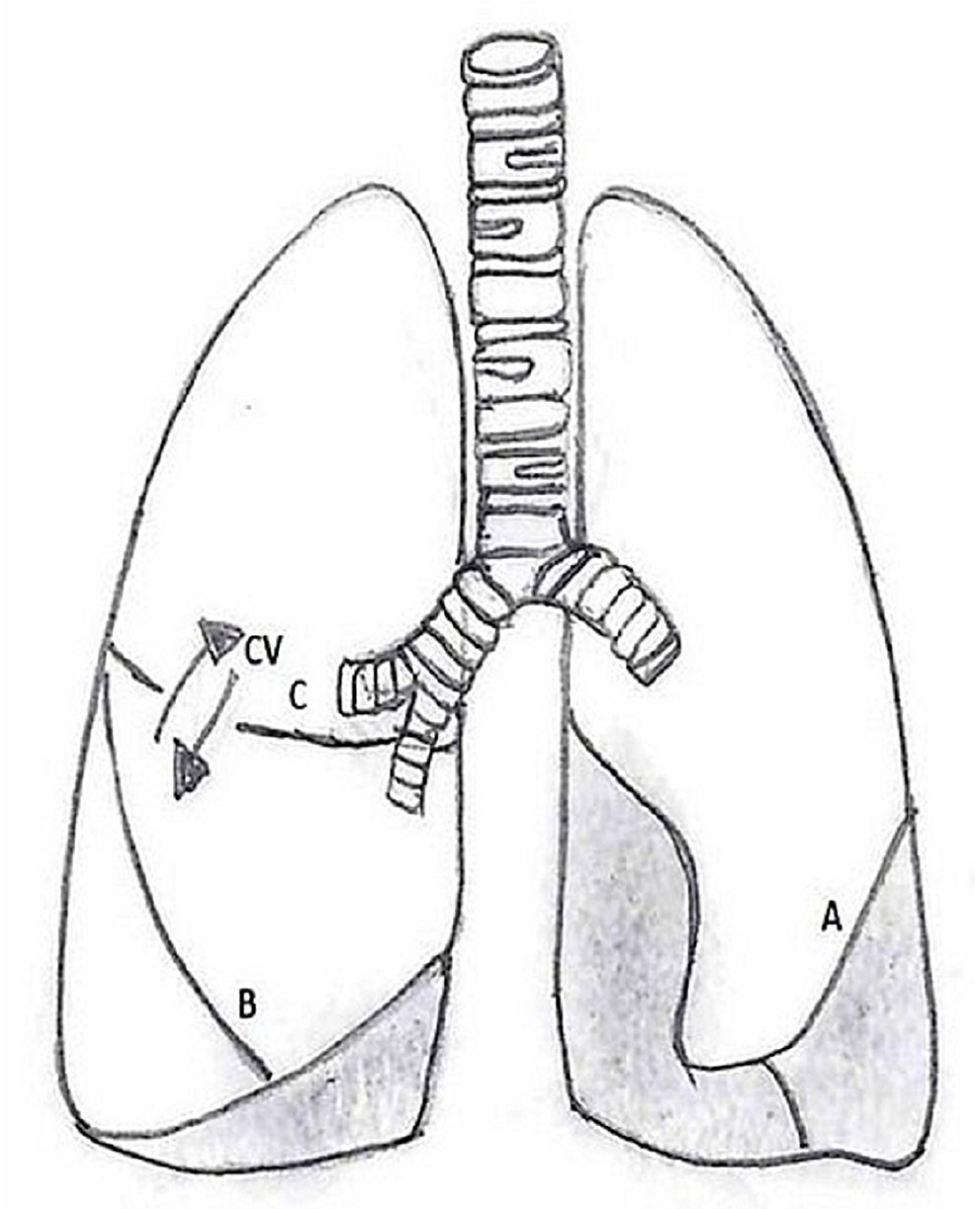
Line diagram showing fissures of the lungs with collateral ventilation in the right lung: (A) Left oblique fissure, (B) right oblique fissure, and (C) right horizontal fissure The diagram was made by one of the authors and has been adapted from the published work by Koster and Slebos [5]. CV: Collateral ventilation.

In this study, the fissures of the lungs were categorized based on the following criteria [1]: The fissure that cuts the whole thickness of the lung, lined by visceral pleura except at the hilum, where they are held together by bronchi and pulmonary vessels was considered as the complete fissure. The fissure starting on the mediastinal surface from the upper part of the hilum and intersecting at the vertebral border (fourth/fifth thoracic spine), coursing along the costal surface, cutting the inferior border, re-appearing on the mediastinal surface, and ending at the lower end of the hilum was taken as a complete oblique fissure. The fissure present in the right lung starting from an oblique fissure at the midaxillary line, running horizontally forward to the anterior border of the lung, and then passing backward to the hilum on the mediastinal surface separating the superior and middle lobes was considered as a complete horizontal fissure. If there were areas of parenchymal fusion between the lobes and the cleft that failed to reach the hilum, we considered it as an incomplete oblique/horizontal fissure. Completely fused parenchyma was taken as an absent fissure. Any additional fissure present apart from the usual one was considered as an accessory fissure. After identifying the fissures in all the lungs, photographs of lungs showing variations were taken (Figure 1). 

Statistical methods

Statistical analyses were performed using MS Excel (Microsoft Excel for Mac, version 2020, Microsoft Corporation, Redmond, Washington, United States). Data were entered and the percentage of occurrence of each abnormal fissure was calculated.

## Results

Variations in the fissures of both lungs, as observed during cadaveric dissection, are described as follows. A total of 32 right lungs were studied, and the variations observed are listed in Table 1.

**Table 1:** Lung fissures The table shows the number and percentage of variant pulmonary fissures in both the lungs in the present study.

Details of fissure	Right lung (n = 32)				Left lung (n = 38)	
	Oblique fissure		Horizontal fissure		Oblique fissure	
	Sternocostal surface	Mediastinal surface	Sternocostal surface	Mediastinal surface	Sternocostal surface	Mediastinal surface
Complete	22 (68.75%)	20 (62.5%)	20 (62.5%)	20 (62.5%)	26 (68.5%)	26 (68.5%)
Incomplete	06 (18.75%)	08 (25%)	04 (12.5%)	04 (12.5%)	08 (21%)	08 (21%)
Absent	04 (12.5%)	04 (12.5%)	08 (25%)	08 (25%)	04 (10.5%)	04 (10.5%)
Accessory fissure	Nil	Nil	Nil	Nil	Nil	Nil

**Figure 2:**
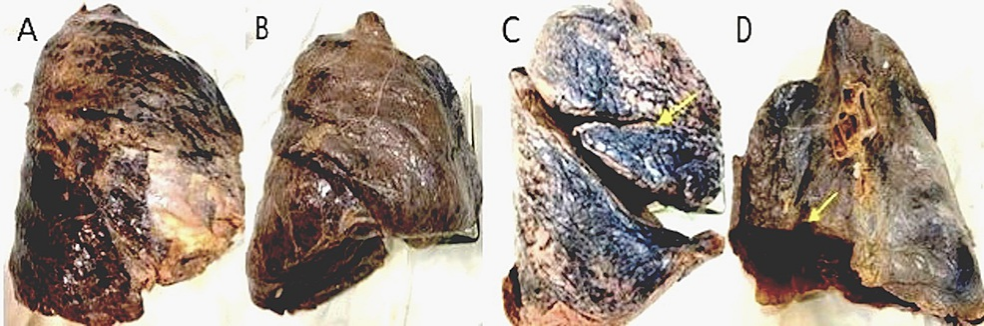
Right lung fissures Variations in fissures of the right lung: (A) Complete absence of fissure on the sternocostal surface, (B) complete absence of horizontal fissure on the sternocostal surface, (C) incomplete horizontal fissure (shown by yellow arrow) on the sternocostal surface, and (D) incomplete oblique fissure (shown by yellow arrow) on the sternocostal surface.

Figure 2 depicts some of the variations observed in the right lungs.

**Figure 3:**
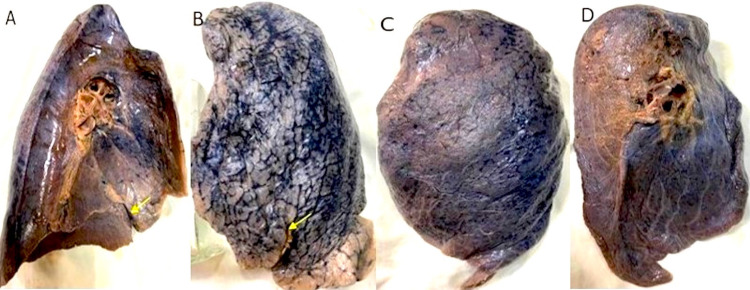
Left lung fissures The variations in fissures of the left lung: (A) Incomplete oblique fissure (shown by yellow arrow) on the mediastinal surface, (B) incomplete oblique fissure (shown by yellow arrow) on the sternocostal surface, (C) absent oblique fissure on the sternocostal surface, and (D) absent oblique fissure on the mediastinal surface.

In addition, 38 left lungs were also studied, and the variations noted are enumerated in Table 1, and some of the variations are shown in Figure 3.

## Discussion

The fissures divide the lungs into lobes and help in the uniform expansion of this vital organ. Any variation in the architecture of the fissures may change the lobar and segmental patterns of the lung. Lobes that remain intact at the hilum by bronchi and pulmonary vessels suggest a complete fissure, whereas the occurrence of parenchymal fusion between lobes suggests an incomplete fissure [4]. Lung fissures develop in intrauterine life, approximately four weeks after conception when the airways start to develop. The lungs develop as a respiratory diverticulum from the cranial foregut, which afterward differentiates into various components of the respiratory tree and parenchyma. During fetal development, the coelomic cavity is divided into the pericardial, pleural, and peritoneal cavities. Pleura, a mesothelial membrane, lines the pleural cavity. As the lung buds grow into the left and right pleural cavities, they are covered by this serous membrane. The portion of the membrane covering the lung buds becomes the visceral pleura, and the portion lining the body wall becomes the parietal pleura. Eventually, all the spaces between individual bronchopulmonary segments get obliterated, except along the lines of division of the principal bronchi, where a deep complete fissure lined by visceral pleura remains patent. This fissure divides the right lung into three lobes and the left lung into two lobes. If the visceral pleura does not cover the lobe completely, lobes can be fused partially or completely, thereby forming an “incomplete fissure” or “absent fissure.” As the pleura invaginates laterally, the fissures are frequently considered incomplete near the hilum [7].

The anatomy of fissures and their completeness have been studied by CT and also on postmortem cases and cadavers. In the past two decades, several studies were conducted by radiologists to recognize fissure completeness, but the incidence of incomplete fissures remains controversial. In the radiological studies, the reported prevalence of incomplete fissures is 17%-85%, 19%-74%, and 20%-90% for the right oblique, left oblique, and horizontal fissures, respectively [8-11]. These studies steadily indicate that the incidence of incomplete minor fissures is more common and is located more frequently near the hilum. Incomplete fissures were also observed in normal healthy individuals [8,12]. In some autopsy studies, incomplete fissures were very common, accounting for 70% in the right major fissure (mainly superior), 46% across the left major fissure (mainly inferior), and 94% across the minor fissures [13]. Extra fissures in the lungs have also been observed by some researchers [14].

Cadavers are still the best source to study human anatomy. In this study, the incidence of incomplete oblique fissure in the right lung was 18.75% on the sternocostal surface and 25% on the mediastinal surface, whereas the incidence in the left lung was 21% on both surfaces. In previous studies, the incidence of incomplete oblique fissures was considerably varied between the right and left lungs (5.3%-61% and 18%-52%, respectively) (Table 2). Regarding fissure absence, our study found a completely absent oblique fissure in 12.5% and 10.5% of the right and left lung specimens, whereas other studies found it in 3%-12% and 4%-10.7%, respectively. Sudikshya et al. [15] did not report absent oblique fissures in18 any of their specimen/lungs.

Furthermore, we found an incomplete horizontal fissure in 12.5% of the right lung samples on both the sternocostal and mediastinal surfaces. In previous studies, its incidence was widely varied, ranging from 21% to 83%. The horizontal fissure was absent in 25% of all lungs in our study, close to the findings of Bergman et al. [16]; however, its incidence was mostly between 6.6% and 16.6%. The highest incidence was reported by Dutta et al. (34.62%) [17], while Mamatha et al. [18] did not find horizontal fissure absence in any of the lungs.

Incomplete oblique fissure is equally prevalent in both lungs in our study, similar to the observation of Bergman [16] and Prakash et al. [19]. However, its incidence is high in the left lung in some studies [13,15] and the right lung in other studies [17,18,20,21]. The prevalence of absent oblique fissure is comparatively low compared to that of absent horizontal fissure in our study, consistent with other reports, except those of Prakash et al. [19] and Mamatha et al. [18].

The lung fissures are usually used as a landmark in specifying pulmonary lesions, and its completeness is critical in planning the treatment for pulmonary diseases [22]. The results of the present study in comparison with the previous works confirmed that incomplete fissures can also occur in healthy individuals and that their prevalence is substantially varied among different populations. Therefore, various genetic and environmental factors might affect the development of these fissures. The incomplete fissure may be the source of collateral ventilation, which refers to the ventilation of alveolar structures through passages that bypass the normal airways. Endobronchial lung volume reduction therapy is promising and proven effective for selected patients with emphysema with planned atelectasis in a particular lung lobe. However, this therapy is contraindicated to those with incomplete or absent fissures because of the existence of collateral ventilation. Therefore, only patients with complete fissures can receive such therapy [5]. This study also unfolds that parenchymal fusion of various extents is a very common entity that may require more lung parenchymal dissection to reach the bronchi and pulmonary arteries during lung resections and thoracoscopic surgeries to avoid perioperative hemorrhage and more postoperative complications, including air leak. Documentation and familiarization of these anomalies remain important to make correct radiological diagnoses for proper surgical management of lung pathology. Recognition of lung anomalies improves our understanding of pneumonia, pleural effusion, and collateral air drift along with the disease spreading through the lungs. Findings from previous studies using cadaveric lung samples are enumerated in Table 2.

**Table 2:** Incidence of pulmonary fissures observed by various authors in the cadaveric studies

Authors	Right lung				Left lung		
	Oblique fissure		Horizontal fissure			Oblique fissure	
	Incomplete	Absent	Incomplete	Absent		Incomplete	Absent
Sudikshya et al., 2018 [15]	30.43	-	34.78	13.04		51.85	-
Mamatha et al., 2016 [18]	50	0	50	0		35	5
Manoj et al., 2014 [20]	14	4	28	8		18	4
Jacob and Pillay, 2013 [21]	50	3.4	83.4	6.6		38.9	-
Dutta et al., 2013 [17]	61.54	11.5	38.89	34.62		48	8
Prakash et al., 2010 [19]	39.3	7.1	50	7.1		35.7	10.7
Bergman et al., 2008 [16]	30	-	67	21		30	0
Present study	22%	12.5%	12.5%	25%		21	10.5%

## Conclusions

Incompleteness and absence of horizontal and oblique fissures were observed in this study, which either agree or disagree with the previous findings. The incidence of incomplete oblique fissures is the same in both lungs. Furthermore, the incidence of absent horizontal fissures is more common than that of absent oblique fissures. Knowledge of such variations in lung fissures helps elucidate the confusing presentation of certain clinical cases of lung pathologies. In addition, knowing the frequency of occurrence of fissure variations in a particular population might help surgeons in planning, executing, and modifying their surgical procedures accordingly as well as radiologists in making correct diagnoses.
